# *In vivo* growth of retinoblastoma in a newborn infant

**DOI:** 10.4103/0301-4738.67066

**Published:** 2010

**Authors:** Parag K Shah, V Narendran, N Kalpana

**Affiliations:** Pediatric Retina and Ocular Oncology Department, Aravind Eye Hospital & Postgraduate Institute of Ophthalmology, Coimbatore, Tamil Nadu, India

**Keywords:** Half-dose carboplatin, growth measure, *in vivo*, retinoblastoma

## Abstract

Retinoblastoma is a rare malignancy of the retina seen exclusively in children. It is known to cause rapid growth inside the eye and hence treatment should be started as soon as it is diagnosed. We report a case in a five-day-old infant in whom treatment (chemotherapy) was delayed by a month due to high bilirubin levels secondary to physiological jaundice, which gave us the unique opportunity to measure the growth of the tumor over a month. This case emphasizes that immediate treatment is warranted once this rare disease is diagnosed.

Retinoblastoma is the commonest intraocular malignancy of childhood.[1] It has been shown to grow rapidly in the eye.[2] Uncontrolled growth can lead to not only destruction of the eye but also extraocular metastasis. There has been only one report[2] where they have measured the growth of the tumor over 13 days in a preterm newborn who was diagnosed to have retinoblastoma and was waiting for brachytherapy. We report a case of a full-term newborn with bilateral disease, where we had the opportunity to observe the growth of the tumor in one eye over 35 days, while the child was waiting for the physiological jaundice to come down in order to start full-dose systemic chemotherapy.

## Case Report

A five-day-old newborn, with a positive family history of retinoblastoma was referred to our institute with a possibility of the same in left eye (LE). On fundus examination right eye (RE) showed three small tumors (< 1 mm in size), two above the supero-temporal arcade vessels and one nasal to the disc. LE showed a solid elevated mass over the macula. The horizontal diameter of that mass was 5.8 mm; vertical diameter was 5.6 mm while the height on B scan was 3.1 mm [Figs. 1a and 1b]. The area of the tumor base was 24.5 mm^2^ and it was 0.24 mm from the temporal margin of the optic disc. A smaller mass with horizontal diameter of 1.7 mm and vertical diameter of 1.5 mm was also present touching the main mass in the supero-temporal quadrant. Transpupillary thermotherapy (TTT) was applied to all the three tumors in RE while systemic chemotherapy was decided for the LE. Unfortunately, on referring to our oncologist, the child was found to have physiological jaundice with the bilirubin levels at 22 mg/dl. The child was subjected to phototherapy and chemotherapy was deferred by two weeks. On Day 16 the bilirubin levels were still high at 5.7 mg/dl and the tumor size in LE had increased. Now, the horizontal diameter was 7.2 mm, vertical was 6.8 mm, height was 3.5 mm [Figs. 2a and 2b] and basal area was 39.8 mm^2^. The tumor was now touching the temporal disc margin. The smaller mass was almost fused with the main tumor. Another small tumor (< 1 mm in diameter) was seen nasal to disc for which TTT was done. Brachytherapy was deferred as there was more than one mass and it would take two to three weeks for the iodine 125 (I_125_) seeds to be prepared. Moreover our brachytherapy experience was limited, especially in newborns. To start some form of treatment immediately, we decided to give half-dose carboplatin. The normal dose is 18.6 mg/kg. We administered 9.3 mg/kg. The tumors in the RE were scarred. Two weeks after the half-dose carboplatin (Day 35 from initial diagnosis) was started the horizontal diameter had increased to 8.7 mm, vertical to 7.8 mm, height to 5.6 mm [Figs. 3a and 3b] and basal area to 55.7 mm^2^. The tumor was now overlapping the temporal disc margin. The nasal tumor had scarred post TTT laser. Fortunately, at that point the bilirubin levels had become normal and full-dose three-drug chemotherapy (carboplatin, etoposide, vincristine) was started immediately. The tumor responded well and after completing six cycles of chemotherapy, at six-month follow-up, it had regressed into a calcified mass (Type 1 regression). This delay in starting the treatment gave us the unique opportunity to study the growth of the tumor over a month *in vivo*.

**Figure 1a:**
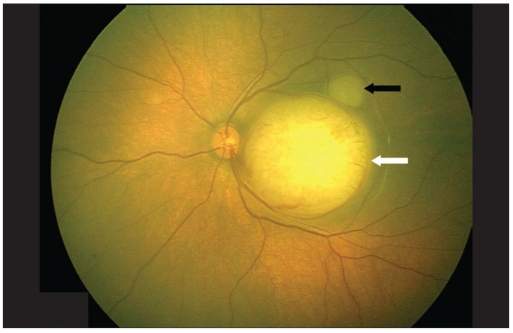
Retcam photo of left eye fundus on Day 0 showing a large macular tumor (white arrow) along with a smaller one touching the main mass in superotemporal quadrant (black arrow). Note the macular tumor is 0.24 mm away from the temporal optic disc margin

**Figure 1b:**
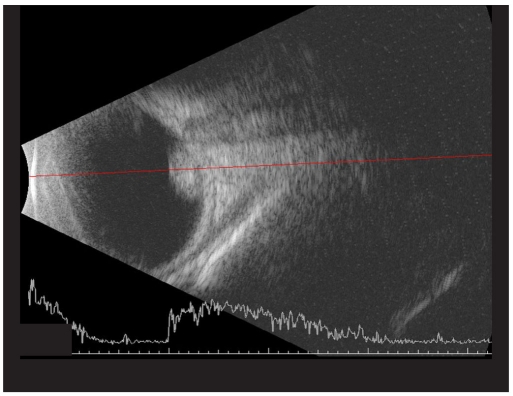
B scan picture of same eye showing the mass on Day 0

**Figure 2a:**
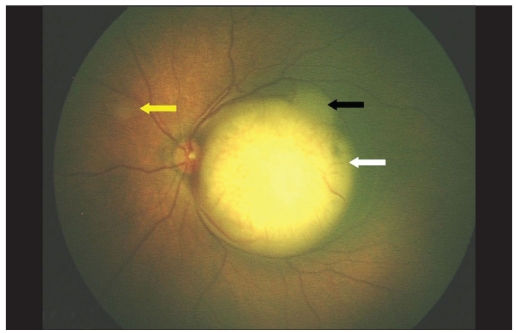
Retcam photo of the same eye on Day 16 showing increased size in the macular tumor (white arrow) while the smaller superotemporal mass is almost fused with the macular mass (black arrow). A smaller tumor nasal to disc (yellow arrow) is also seen. Note that the macular tumor is now touching the temporal disc margin

**Figure 2b:**
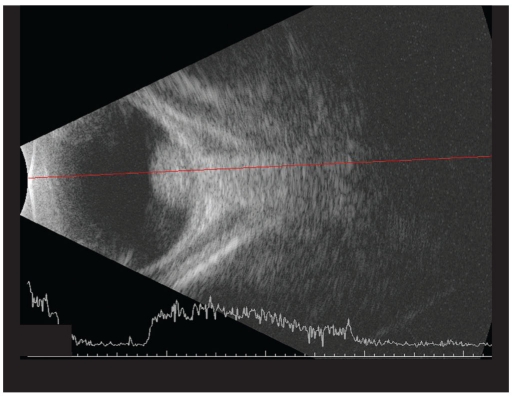
B scan picture of the same eye on Day 16 showing the mass increased in size

**Figure 3a:**
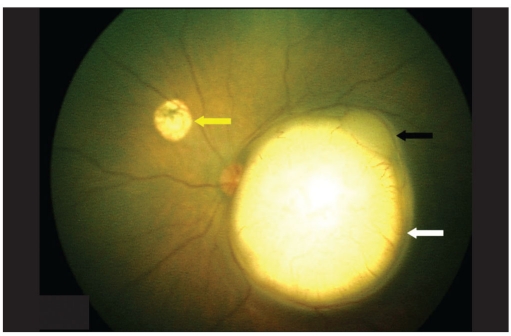
Retcam photo on Day 35 showing increased size of macular mass (white arrow) compared to Day 16 photo and the fused smaller superotemporal mass (black arrow). Yellow arrow nasal to optic disc shows the scarred tumor post transpupillary thermotherapy laser. Note that the macular tumor is now overlapping the temporal disc margin

**Figure 3b:**
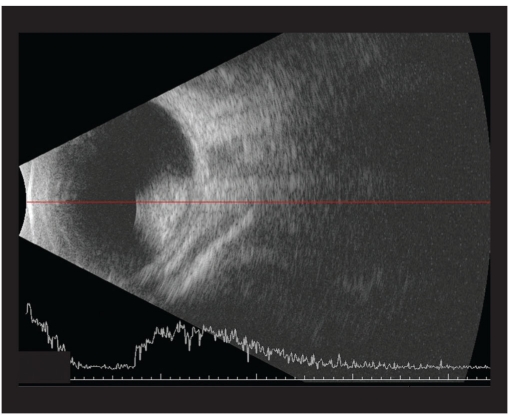
B scan picture taken on Day 35 showing a larger mass

## Discussion

In India, survival rates of patients with retinoblastoma are getting better. This is observed by the fact that now we are seeing the second generation of the survivors of germinal type of retinoblastoma. The child’s father was also one such survivor and hence the tumor was picked up in this child very early in life. In spite of being diagnosed early (at five days of life), immediate chemotherapy could not be started because of physiological jaundice. Brachytherapy although available at our center, was not utilized due to our inexperience in doing it in newborns and also it would have taken at least two to three weeks for the I_125_ seeds to be prepared. Moreover, there were multiple tumors. On second follow-up at Day 16 when the bilirubin levels were still high, we decided to give half-dose carboplatin (9.3 mg/kg instead of 18.6 mg/kg). However, this did not work and by Day 35 the tumor volume had grown about four times. Volume of the tumor was calculated using the formula for ellipsoid 4/3 ∏ abc where ∏ = 22/7, a and b are radii of basal diameters and c is radius of height. Basal diameters were measured on Retcam images using the software while the height was measured by B scan imaging. Area of tumor base was also calculated on Retcam images using the software. Fortunately, full-dose chemotherapy could be started as the bilirubin levels had returned to normal by that time.

This delay in treatment gave some time to study the tumor growth. On Day 16 there was a 1.7 times increase in tumor volume and by Day 35 there was 3.9 times increase. We agree with Abramson *et al*.[2] that the tumor doubling time for retinoblastoma must be around 15 days. However, as they mentioned, we also urge caution in assuming that 15 days is the doubling time for all retinoblastomas since the child was very young and was also treated with half-dose carboplatin midway. Phase transit time in the retinal progenitor cells in this young child is likely to be shorter.[2] In general, in young developing organisms only those tumors with very short doubling time are likely to exist and survive, where as, later during a period of weakening cell-cell cooperation, cancer types with longer doubling times can thrive.[2,3]

Thus the clinical measure of this rapid growth is important and emphasizes the need to start immediate treatment once retinoblastoma is diagnosed.
